# U. S. Signal Service

**Published:** 1877-11-20

**Authors:** 


U. S. SIGNAL SERVICE.—At Fort Whipple, Virginia, the Government has established a school of instruction for those who are to engage in the Signal Service. The parties in attendance are mostly connected with the army, and are taught military signaling, telegraphy and meteorological science. There are now in the United States 171 Stations for making and reporting observations. There are also, several stations in the West Indies, and in British America.

The "synopsis of Probabilities," which is made at the Central Station in Washington, which, in the newspapers, have received the *soubriquet* of "Old Probs," has, by its constantly increasing accuracy, secured the confidence of the people, and led to the hope that almost absolute certainty in weather predictions will ultimately be attained.

| | | | | |
|---|---|---|---|---|
| In 1872 | the percentage | of verifications | was | 76.8 |
| " 1873 | " | " | " | 77.6 |
| " 1874 | " | " | " | 84.4 |
| " 1875 | " | " | " | 87.4 |
| " 1876 | " | " | " | 88.3 |

While these observations were orignally instituted for the benefit of commerce and agriculture, it is evident, when we consider the susceptibility of the human system to meterological conditions, that they well deserve the attention of medical men, and should be studied with reference to their bearing upon health and disease. W.

